# Highly diverse and unknown viruses may enhance Antarctic endoliths’ adaptability

**DOI:** 10.1186/s40168-023-01554-6

**Published:** 2023-05-08

**Authors:** Cassandra L. Ettinger, Morgan Saunders, Laura Selbmann, Manuel Delgado-Baquerizo, Claudio Donati, Davide Albanese, Simon Roux, Susannah Tringe, Christa Pennacchio, Tijana G. del Rio, Jason E. Stajich, Claudia Coleine

**Affiliations:** 1 Department of Microbiology and Plant Pathology, University of California, Riverside, CA USA; 2 Department of Biological Sciences, California State Polytechnic University, Pomona, CA USA; 3 The University of Arizona, Tucson, AZ USA; 4 Department of Ecological and Biological Sciences, University of Tuscia, Viterbo, Italy; 5 Laboratorio de Biodiversidad Y Funcionamiento Ecosistémico, Instituto de Recursos Naturales Y Agrobiología de Sevilla (IRNAS), CSIC, Av. Reina Mercedes 10, Seville, E-41012 Spain; 6 Unidad Asociada CSIC-UPO (BioFun), Universidad Pablo de Olavide, Seville, 41013 Spain; 7 Research and Innovation Centre, Fondazione Edmund Mach, Via E. Mach 1, San Michele All’Adige, 38098 Italy; 8 Department of Energy Joint Genome Institute, Lawrence Berkeley National Laboratory, One Cyclotron Road, Berkeley, CA 94720 USA; 9 Institute for Integrative Genome Biology, University of California, Riverside, CA USA

## Abstract

**Background:**

Rock-dwelling microorganisms are key players in ecosystem functioning of Antarctic ice free-areas. Yet, little is known about their diversity and ecology, and further still, viruses in these communities have been largely unexplored despite important roles related to host metabolism and nutrient cycling. To begin to address this, we present a large-scale viral catalog from Antarctic rock microbial communities.

**Results:**

We performed metagenomic analyses on rocks from across Antarctica representing a broad range of environmental and spatial conditions, and which resulted in a predicted viral catalog comprising > 75,000 viral operational taxonomic units (vOTUS). We found largely undescribed, highly diverse and spatially structured virus communities which had predicted auxiliary metabolic genes (AMGs) with functions indicating that they may be potentially influencing bacterial adaptation and biogeochemistry.

**Conclusion:**

This catalog lays the foundation for expanding knowledge of virosphere diversity, function, spatial ecology, and dynamics in extreme environments. This work serves as a step towards exploring adaptability of microbial communities in the face of a changing climate.

Video Abstract

**Supplementary Information:**

The online version contains supplementary material available at 10.1186/s40168-023-01554-6.

## Background

Viruses are among the most prevalent entities on our planet, with the ability to infect organisms across all domains [1]. Sequencing advances are reshaping understanding of viral diversity across Earth’s diverse ecosystems, leading to a remarkable expansion of viral catalogs[1–6]. It is becoming clear that viruses play key roles in global biogeochemical cycles through the modulation of host population dynamics, and that the better-studied pathogenic viruses represent only a small fraction of the virosphere [7–9]. Further, through auxiliary metabolic genes (AMGs), some viruses can directly impact host metabolism to improve fitness [10], including in terrestrial ecosystems characterized by extreme conditions (e.g., oligotrophy, aridity, high, or low temperature).

Antarctic ice-free areas include several of the most inhospitable regions on Earth, among which is the Mars counterpart [11]: the McMurdo Dry Valleys. In these locations, where rocks represent the main substratum, active life is possible for only a few specialized microorganisms; they survive by dwelling in porous rocks, forming self-sustaining ecosystems called endolithic communities [12, 13]. These microorganisms are the primary life-forms present assuring the balance and functionality of these otherwise inert ecosystems. Recent studies have shed light on their biodiversity and adaptation, particularly the evolution of new and peculiar taxa spanning bacteria, fungi, and archaea [13–16]. However, the ecology and distribution of viral diversity from these communities remain wholly unknown and, to date, viral studies have instead focused on Antarctic freshwater lakes [17–19], surrounding oceans [20–22], and soils [23–26].

Here, we provide a large-scale viral catalog from 191 Antarctic endolith metagenomes. We sampled 37 localities across a broad range of environmental (e.g., 4 rock typologies, different altitudes and sun exposure) and spatial conditions (i.e., Antarctic Peninsula, Northern Victoria Land, and McMurdo Dry Valleys) (Table S1; Fig. 2A). We aimed to (i) untangle viral diversity in these communities, (ii) predict AMGs and how they may drive the fitness of their hosts, and (iii) explore ecological patterns (e.g., biogeography). This catalog is the first step toward understanding the role of viruses in the coldest and driest region on Earth. This information is also critical for elucidating the possible role of viruses in whole community adaptation in a dry ecosystem that will expand owing to global change [27].

## Methods

### Study area

One hundred ninety-one rocks colonized by endolithic communities were collected in thirty-eight sites in Antarctica including Antarctic Peninsula (*n* = 3), McMurdo Dry Valleys, Southern Victoria Land (*n* = 80), and Northern Victoria Land (*n* = 108) during more than 20 years of Italian Antarctic Expeditions. Different rock typologies (sandstone *n* = 141, granite *n* = 43, quartz *n* = 5, and basalt/dolerite *n* = 2) were sampled. Samples were collected along a latitudinal transect ranging from − 62.10008 − 58.51664 to − 77.874 160.739 at different environmental conditions namely sun exposure (northern sun exposed and southern shady rocks) and an altitudinal transect from sea level to 3100 m above sea level (a.s.l.) to provide a comprehensive overview of Antarctic endolithic diversity (Table S1). The presence of endolithic colonization was assessed by direct observation in situ. Rocks were excised using a geologic hammer and sterile chisel, and rock samples were preserved in sterile plastic bags, transported, and stored at – 20 °C in the Culture Collection of Antarctic fungi of the Mycological Section of the Italian Antarctic National Museum (MNA-CCFEE), until downstream analysis.

### Study data

In total, the dataset included 191 metagenomes, of which 100 have been assembled as described in Albanese et al. [14]. The remaining metagenomes were generated, sequenced, and assembled as described below. The final metagenomic set represented 149,585,625 metagenomic contigs.

### DNA extraction, library preparation, and sequencing

Total community DNA was extracted from 1 g of crushed rocks using DNeasy PowerSoil Pro Kit (Qiagen, Germany), quality checked by electrophoresis using a 1.5% agarose gel and Nanodrop spectrophotometer (Thermofisher, USA) and quantified using the Qubit dsDNA HS Assay Kit (Life Technologies, USA) according to Coleine et al. [13]. Shotgun metagenomic sequencing paired-end libraries were constructed by using Next Ultra DNA library prep kits and sequenced as 2 × 150 bp using the Illumina NovaSeq platform (Illumina Inc., San Diego, CA, USA) at the Edmund Mach Foundation (San Michele all’Adige, Italy) and at the DOE Joint Genome Institute (JGI).

### Sequencing reads preparation and assembly

The metashot/mag-illumina v2.0.0 workflow (https://github.com/metashot/mag-illumina, parameters: –metaspades_k 21,33,55,77,99) was used to perform raw reads quality trimming and filtering, and assembly on the metagenomic samples. In brief, adapter trimming, contaminant (artifacts and and spike-ins) and quality filtering were performed using BBDuk (BBMap/BBTools v38.79, https://sourceforge.net/projects/bbmap/). During the quality filtering procedure i) raw reads were quality-trimmed to Q6 using the Phred algorithm; ii) reads that contained 4 or more “N” bases, had an average quality below 10, shorter than 50 bp or under 50% of the original length were removed. Samples were then assembled individually with SPAdes v3.15.1 [28] (parameters –meta -k 21,33,55,77,99).

### Identification and clustering of viral genomes

Using a workflow similar to Guo et al. [29], viral sequences were identified separately for each of the 191 metagenomic assemblies using VirSorter2 v. 2.2.3 [30] using –min-length 5000, –min-score 0.5, and –include-groups dsDNAphage,NCLDV,RNA,ssDNA,lavidaviridae. CheckV v0.8.1 [31] was run on the VirSorter2 predicted viral sequences using the “end_to_end” workflow VirSorter2 was then run again on the viral sequences from CheckV workflow with the –prep-for-dramv option. DRAM-v v. 1.2.2 [32] was then used to “annotate” sequences against databases downloaded on August 3rd, 2021 including Viral RefSeq, KOfam [33], Pfam [34], dbCAN v.9 [35], MEROPS [36], and VOGDB (http://vogdb.org/). DRAM-v was then used to “distill” annotations into predicted AMGs for phage.

Viral sequences from all assemblies were combined and clustered into 95% similarity viral operational taxonomic units (vOTUs) using CD-HIT v. 4.8.1 [37] with the following parameters: -c 0.95 -aS 0.85 -M 0 -d 0. Prodigal v. 2.6.3 [38] was used to predict open reading frames in vOTUs using the -p meta option. VContact2 v. 0.9.19 was then run on predicted proteins from phage vOTUs and predicted proteins from the INPHARED August 2022 viral reference database to generate viral clusters (VCs) based on gene-sharing networks [39, 40]. We assigned taxonomy to phage vOTUs based on VC membership as in Santos-Medellin et al. [41]. Predicted viral sequences and 95% similarity vOTUS are archived on Zenodo [42].

### Viral host-prediction

Hosts were predicted for the phage sequences identified using (i) a database of complete genomes from NCBI RefSeq, and (ii) a previously published database of representative metagenome-assembled genomes (MAGs) from Antarctic endolith samples. To produce (i), we used “ncbi-genome-download” to download all complete bacterial (*n* = 25,984) and archaeal (*n* = 416) genomes, as of April 7, 2022, from NCBI RefSeq [43]. For (ii), we downloaded MAGs from Zenodo (https://doi.org/10.5281/zenodo.7313591). We then used NCBI BLAST 2.12.0 + to convert these two databases into blast databases using “makeblastdb” and used “blastn” to compare vOTUs to these databases [44]. We filtered the blastn results in R based on existing thresholds [45–47]. Briefly, database matches had to share ≥ 2000 bp region with ≥ 70% sequence identity to the viral sequence and needed to have a bit score of ≥ 50 and minimum *e* value of 0.001. Further to ensure matches did not represent partial or entirely viral contigs when searching against the MAG database, matches had to cover < 50% of the total MAG sequence length. As in Korthari et al. [46], only the top 5 hits matching these thresholds were considered, with host predictions made at each taxonomic level only if the taxonomy of all hits were in agreement. Discrepancies resulted in no host prediction for that taxonomic level. We then combined host predictions from both the RefSeq and MAG databases together; if there were discrepancies between the two databases, we defaulted to the MAG-based prediction.

### Ecological analysis of vOTUs

We mapped reads from each metagenome to vOTUs using BBMap with a minimum sequence similarity of 90% to quantify vOTU relative abundance [48]. We then used SAMtools to convert resulting sam files to bam files and genomecov from BEDTools to obtain coverage information for each vOTU across each metagenome [49, 50]. We then used bamM to parse bam files and calculate the trimmed pileup coverage (tpmean), which we used here in our analysis of viral relative abundance [51]. We removed vOTUs which displayed < 75% coverage over the length of the viral sequence and viral sequences < 10 kbp in length prior to downstream analyses in R [52]. Thresholds for analysis of vOTUs were based on community guidelines for length (i.e., ≥ 10 kbp), similarity (i.e., ≥ 95% similarity), and detection (i.e., ≥ 75% of the viral genome length covered ≥ 1 × by reads at ≥ 90% average nucleotide identity) [53, 54]. To be conservative, we also removed vOTUs with a CheckV quality score of “not-determined” prior to downstream analysis. The viral abundance (tpmean), quality, taxonomy, and annotation results were imported, analyzed, and visualized in R using many packages including tidyverse and phyloseq [55, 56]. Analysis scripts associated with this study are on GitHub and archived in Zenodo [57].

To compare viral diversity between metagenomes (i.e., beta diversity), we calculated the Hellinger distance, the Euclidean distance of Hellinger transformed abundance data. We performed Hellinger transformations using the transform function in the microbiome R package, calculated the Hellinger distance using the ordinate function in phyloseq, and then visualized these distances using principal-coordinate analysis (PCoA). We performed permutational multivariate analyses of variance (PERMANOVAs) with 9,999 permutations to test for significant differences in mean centroids using the model: Distance ~ Site + Rock type. Models were tested with “by = margins” and “by = terms” with all sequential combinations. We ran the ordistep and ordiR2step functions to help assess optimal parameters to include in the model. Since PERMANOVA tests are sensitive to differences in group dispersion, we also tested for significant differences in mean dispersions using the betadisper and permutest functions from the vegan package in R with 9,999 permutations.

To test for correlations between viral community distances (Hellinger distances) and geographic distances, we first subset the data to exclude metagenomes from the Antarctic Peninsula, and to account for variation between rock types, subset the data to include only metagenomes representing sandstone samples. We calculated geographical distances between metagenomes using the distm function in the geosphere package in R. We performed Mantel tests in the vegan R package to assess correlations between the community and geographic distances using a Spearman correlation and 9999 permutations. Mantel tests were repeated with exclusion of community distances when the geographic distance was zero to assess if patterns persisted in the absence of data from the same site.

## Results and discussion

Using VirSorter2 [30], we predicted 101,085 viral sequences. We clustered these at 95% average nucleotide identity into 76,984 vOTUS [37]; we further used VContact2 [39] with INPHARED [40] reference genomes to cluster phage vOTUs into 7598 VCs, which approximate genus-level groupings based on gene-sharing networks. To keep analysis focused on the most robust catalog, we filtered this collection using community thresholds for length, detection, and quality (see “Methods” section) [31, 53, 54]. The final viral catalog represented 14,796 viral sequences (Table S2; 76 complete, 341 high-quality, 1539 medium-quality, 12,840 low-quality), including 2,695 prophage, which clustered into 11,806 vOTUs, of which 5743 phage vOTUs (7309 sequences) were successfully placed in 2286 VCs; the final catalog was predicted to predominantly be dsDNA phage, though 15.2% of vOTUs may represent eukaryotic viruses (i.e. nucleocytoplasmic large DNA viruses).

**Fig. 1:**
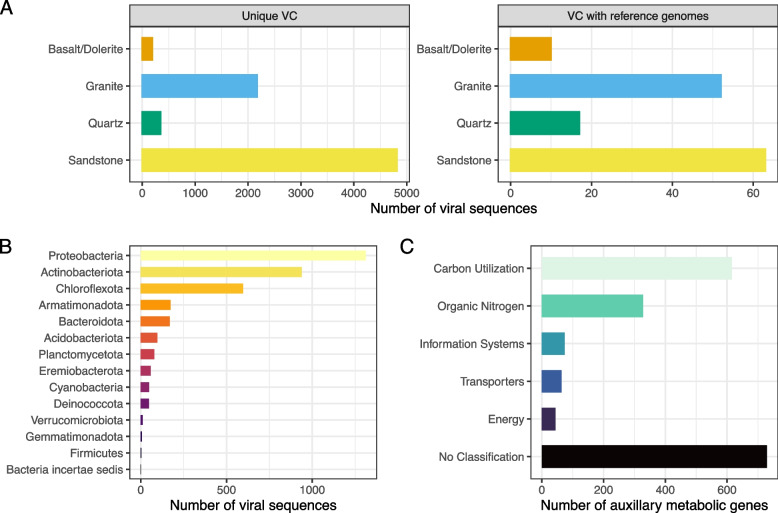
Antarctica is an underappreciated source of phage novelty. **A** Bar charts displaying the number of viral sequences placed in VCs colored by rock type (sandstone *n* = 141, granite *n* = 43, quartz *n* = 5, and basalt/dolerite *n* = 2) and divided by whether the VC is clustered with reference genomes. **B** Bar chart displaying host predictions colored by predicted host phylum. **C** Bar chart showing the number of predicted phage AMGs summarized by DRAM-v distilled metabolic categories

Our findings may indicate that Antarctic rock communities host highly diverse and novel phage populations, with only 1.8% (41 out of 2,286) of the VCs including reference sequences. The remaining 98.2% were unique VCs (i.e., did not include reference genomes), and could represent novel phage genera, greatly expanding the known diversity of viruses. Of the 41 VCs that did include reference genomes, the majority were assigned to the *Caudoviricetes* class (formerly *Caudovirales* order) of tailed double-stranded DNA bacteriophage (Figure S1). Many genomes have not yet been reclassified, leaving viral taxonomy in flux; under the new schema, most of the 41 VCs are unclassified [58]. The majority of unique VCs are represented by viral sequences from sandstone communities (Fig. 1A), which represents an optimum substratum, in terms of rock traits (e.g., porosity), for endolithic colonization [59], but is also the most represented substratum in this work.

We further established host–virus linkages using NCBI BLAST against complete bacteria and archaea genomes from RefSeq, and Antarctic endolithic bacterial and archaeal metagenome-assembled genomes (MAGs) (see “Methods” section) [44–46] to explore the potential effects of viruses on host fitness, such as host-cell reprogramming through AMGs [60]. While we were unable to predict hosts for the majority of viral sequences (only 23.94% had a host prediction), we observed that Proteobacteria, Actinobacteriota, and Chloroflexota were the most commonly predicted host phyla (Fig. 1B), which are thought to be core members of these communities [14, 61, 62]. Using predictions against the Antarctic MAGs, we predicted hosts for an additional 16.5% of viral sequences compared to the 7.48% predicted using RefSeq alone (Figure S2).

We then sought to improve understanding on the functional profiles of retrieved phages using DRAM-v (Table S3) [32]. Notably, this catalog, which comprises metabolic novelty (39.3% of DRAM-v predicted AMGs had no distilled classification), may complement other available resources, which have largely been limited to coverage of human-related microbiomes (e.g. Li et al. [63]). Within identified functions, we found putative phage AMGs related to carbon, energy, and nitrogen metabolisms (Fig. 1C). Specifically, within carbohydrate metabolism, glycoside hydrolases, glycosyltransferases, and carbohydrate-binding domains predominated. Within nitrogen metabolism, methionine degradation was the most prevalent module, and within energy, the dominant modules were related to electron transport and photosynthesis. This highlights the need to connect vOTUs to Antarctic MAGs [14] and to implement complementary techniques (e.g., single-cell genomics) to provide a deeper understanding of virus-bacteria dynamics. More importantly, these findings suggest a possible complex role for viruses in element biogeochemical cycles in the rocks of Antarctica, which have traditionally been considered devoid of life.

**Fig. 2:**
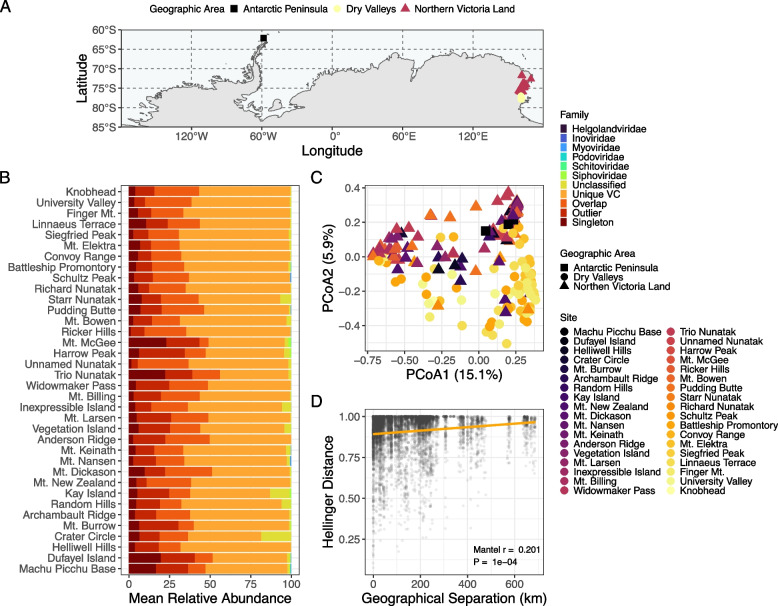
Spatial structuring of viral communities in Antarctic rocks. **A** Map showing collection sites with shapes and colors representative of the broad geographic area. **B** Stacked bar charts displaying the mean relative abundance of phage vOTUs at each site colored by predicted viral families. Sequences that were clustered into VCs with reference data are labeled by their taxonomy, sequences clustered without reference genomes are labeled “Unique VC”, while the rest are labeled based on their VContact2 status (i.e., singleton [share few or no genes with other genomes], overlap [share genes with genomes in multiple VCs], or outlier [share genes, but cannot confidently be placed in a VC]). **C** Principal-coordinate analysis (PCoA) visualization of Hellinger distances of viral communities. Samples are colored by site, with sites ordered by latitude, and have shapes based on geographic areas. **D** A scatter plot depicting a significant relationship between sandstone viral community beta diversity (Hellinger distance) and geographical distance (km) between sites

Given the geographic spread of sampling (see “Methods” section and Table S1; Fig. 2A), we assessed whether this catalog could be useful to answer ecological questions related to viral community dynamics. While the dominant vOTUs at each site were taxonomically unclassified and largely members of unique VCs and thus possible novel genera (Fig. 2B), when investigating between-sample diversity (beta diversity) we observed a significant pattern related to site specificity (Fig. 2C; PERMANOVA, *p* < 0.001). Further, we detected a significant correlation with geographic distance in sandstone communities (Fig. 2D; Mantel *r* = 0.197, *p* < 0.001), such that communities are more dissimilar with increasing distance. Combined these results indicate possible latitudinal spatial structuring of viral communities. In further support of this, we were able to detect only 41.0% of vOTUs at more than one site, with 29.4% of vOTUs detected across two or more geographic regions and only 1.45% detected across all regions. Of the vOTUs detected across all regions, the majority were in unique VCs (66.7%) and none were in VCs with reference data. We hypothesize that this viral spatial structuring reflects the reported dispersion limitation and local composition and adaptation of hosts in these communities [14, 15]. Similar spatial structuring has also been observed in grassland soil viromes, purportedly as a result of local assembly dynamics [41, 64].

## Conclusions

This study represents the most exhaustive geographic endeavor to date to capture the viral genomic diversity across ice-free regions of Antarctica and the first large-scale effort to explore the virosphere in endolithic communities. This catalog is a comprehensive repository for exploring the diversity, function, spatial ecology, and host-virus dynamics of this enigmatic continent. We also unveiled a potential influence of some viruses on carbon, energy, and nitrogen metabolism under conditions of oligotrophy up to the limit for life sustainability. Finally, this work may serve in the future as an important first step towards exploring adaptability of microbial communities in extreme conditions on Earth.

## Supplementary Information

**Additional file 1: Table S1.** Sample information and viral identification statistics from Antarctic metagenomes. **Table S2.** Final viral catalog sequence information. **Table S3.** DRAM-v distilled annotations for predicted phage AMGs. **Figure S1.** Taxonomic classification of viral clusters (VCs) that include reference genomes. **Figure S2.** Comparison of host predictions between MAG and RefSeq databases.

## Data Availability

Metagenomic raw data are available under the NCBI accession numbers listed in Supplementary Table S1. Analysis scripts and intermediate data files associated with this study are on GitHub (https://github.com/stajichlab/Antarctic_Virus_Discovery) and archived in Zenodo (https://doi.org/10.5281/zenodo.7374327). Fasta files representing the entire catalog of predicted viral sequences and 95% similarity vOTUS are archived and available on Zenodo (https://doi.org/10.5281/zenodo.7245811).

## References

[1] Paez-Espino D, Eloe-Fadrosh EA, Pavlopoulos GA, Thomas AD, Huntemann M, Mikhailova N (2016). Uncovering Earth’s virome. Nature.

[2] Emerson JB, Roux S, Brum JR, Bolduc B, Woodcroft BJ, Jang HB, et al. Host-linked soil viral ecology along a permafrost thaw gradient. Nat Microbiol. 2018;3(8):870–80. 10.1038/s41564-018-0190-y PMC6786970 30013236

[3] Gregory AC, Zayed AA, Conceição-Neto N, Temperton B, Bolduc B, et al. Marine DNA viral macro-and microdiversity from pole to pole. Cell. 2019;177(5):1109–23. 10.1016/j.cell.2019.03.040 PMC6525058 31031001

[4] Gregory AC, Zablocki O, Zayed AA, Howell A, Bolduc B, Sullivan MB. The gut virome database reveals age-dependent patterns of virome diversity in the human gut. Cell Host Microbe. 2020;28(5):724–40. 10.1016/j.chom.2020.08.003 PMC7443397 32841606

[5] Roux S, Páez-Espino D, Chen IM, Palaniappan K, Ratner A, Chu K, et al. IMG/VR v3: an integrated ecological and evolutionary framework for interrogating genomes of uncultivated viruses. Nucleic Acids Res. 2021;49(D1):D764–75. 10.1093/nar/gkaa946 PMC7778971 33137183

[6] Neri U, Wolf YI, Roux S, Camargo AP, Lee B, Kazlauskas D, et al. Expansion of the global RNA virome reveals diverse clades of bacteriophages. Cell. 2022;185(21):4023–37. 10.1016/j.cell.2022.08.023 36174579

[7] Kristensen DM, Mushegian AR, Dolja VV, Koonin EV (2010). New dimensions of the virus world discovered through metagenomics. Trends Microbiol.

[8] Jin M, Guo X, Zhang R, Qu W, Gao B, Zeng R (2019). Diversities and potential biogeochemical impacts of mangrove soil viruses. Microbiome.

[9] Hurwitz BL, Westveld AH, Brum JR, Sullivan MB (2014). Modeling ecological drivers in marine viral communities using comparative metagenomics and network analyses. Proc Natl Acad Sci U S A.

[10] Breitbart M, Thompson L, Suttle C, Sullivan M (2007). Exploring the vast diversity of marine viruses. Oceanography.

[11] Cockell CS, Bush T, Bryce C, Direito S, Fox-Powell M, Harrison JP (2016). Habitability: a review. Astrobiology.

[12] Friedmann EI (1982). Endolithic microorganisms in the antarctic cold desert. Science.

[13] Coleine C, Biagioli F, de Vera JP, Onofri S, Selbmann L (2021). Endolithic microbial composition in Helliwell Hills, a newly investigated Mars-like area in Antarctica. Environ Microbiol.

[14] Albanese D, Coleine C, Rota-Stabelli O, Onofri S, Tringe SG, Stajich JE (2021). Pre-Cambrian roots of novel Antarctic cryptoendolithic bacterial lineages. Microbiome.

[15] Archer SDJ, de los Ríos A, Lee KC, Niederberger TS, Craig Cary S, Coyne KJ (2017). Endolithic microbial diversity in sandstone and granite from the McMurdo Dry Valleys, Antarctica. Polar Biol.

[16] de la Torre JR, Goebel BM, Friedmann EI, Pace NR (2003). Microbial diversity of cryptoendolithic communities from the McMurdo Dry Valleys, Antarctica. Appl Environ Microbiol.

[17] López-Bueno A, Tamames J, Velázquez D, Moya A, Quesada A, Alcamí A (2009). High diversity of the viral community from an Antarctic lake. Science.

[18] Prado T, Brandão ML, Fumian TM, Freitas L, Chame M, Leomil L (2022). Virome analysis in lakes of the South Shetland Islands, Antarctica - 2020. Sci Total Environ.

[19] Zawar-Reza P, Argüello-Astorga GR, Kraberger S, Julian L, Stainton D, Broady PA (2014). Diverse small circular single-stranded DNA viruses identified in a freshwater pond on the McMurdo Ice Shelf (Antarctica). Infect Genet Evol.

[20] Alarcón-Schumacher T, Guajardo-Leiva S, Antón J, Díez B (2019). Elucidating viral communities during a phytoplankton bloom on the West Antarctic Peninsula. Front Microbiol.

[21] Miranda JA, Culley AI, Schvarcz CR, Steward GF (2016). RNA viruses as major contributors to Antarctic virioplankton. Environ Microbiol.

[22] Gong Z, Liang Y, Wang M, Jiang Y, Yang Q, Xia J (2018). Viral diversity and its relationship with environmental factors at the surface and deep sea of Prydz Bay, Antarctica. Front Microbiol.

[23] Zablocki O, van Zyl L, Adriaenssens EM, Rubagotti E, Tuffin M, Cary SC (2014). High-level diversity of tailed phages, eukaryote-associated viruses, and virophage-like elements in the metaviromes of antarctic soils. Appl Environ Microbiol.

[24] Adriaenssens EM, Kramer R, Van Goethem MW, Makhalanyane TP, Hogg I, Cowan DA (2017). Environmental drivers of viral community composition in Antarctic soils identified by viromics. Microbiome.

[25] Zablocki O, van Zyl L, Adriaenssens EM, Rubagotti E, Tuffin M, Cary SC (2014). Niche-dependent genetic diversity in Antarctic metaviromes. Bacteriophage.

[26] Bezuidt OKI, Lebre PH, Pierneef R, León-Sobrino C, Adriaenssens EM, Cowan DA (2020). Phages Actively Challenge Niche Communities in Antarctic Soils. mSystems.

[27] Jansson, JK, Wu R. Soil viral diversity, ecology and climate change. Nat Rev Microbiol. 2023;21:296–311. 10.1038/s41579-022-00811-z. 10.1038/s41579-022-00811-z 36352025

[28] Bankevich A, Nurk S, Antipov D, Gurevich AA, Dvorkin M, Kulikov AS (2012). SPAdes: a new genome assembly algorithm and its applications to single-cell sequencing. J Comput Biol.

[29] Guo J, Vik D, Pratama AA, Roux S, Sullivan M. Viral sequence identification SOP with VirSorter2. protocols.io. 2021. https://www.protocols.io/view/viral-sequence-identification-sop-with-virsorter2-btv8nn9w.

[30] Guo J, Bolduc B, Zayed AA, Varsani A, Dominguez-Huerta G, Delmont TO (2021). VirSorter2: a multi-classifier, expert-guided approach to detect diverse DNA and RNA viruses. Microbiome.

[31] Nayfach S, Camargo AP, Schulz F, Eloe-Fadrosh E, Roux S, Kyrpides NC (2021). CheckV assesses the quality and completeness of metagenome-assembled viral genomes. Nat Biotechnol.

[32] Shaffer M, Borton MA, McGivern BB, Zayed AA, La Rosa SL, Solden LM (2020). DRAM for distilling microbial metabolism to automate the curation of microbiome function. Nucleic Acids Res.

[33] Aramaki T, Blanc-Mathieu R, Endo H, Ohkubo K, Kanehisa M, Goto S (2020). KofamKOALA: KEGG Ortholog assignment based on profile HMM and adaptive score threshold. Bioinformatics.

[34] El-Gebali S, Mistry J, Bateman A, Eddy SR, Luciani A, Potter SC (2019). The Pfam protein families database in 2019. Nucleic Acids Res.

[35] Zhang H, Yohe T, Huang L, Entwistle S, Wu P, Yang Z (2018). dbCAN2: a meta server for automated carbohydrate-active enzyme annotation. Nucleic Acids Res.

[36] Rawlings ND, Barrett AJ, Bateman A (2010). MEROPS: the peptidase database. Nucleic Acids Res.

[37] Li W, Godzik A (2006). Cd-hit: a fast program for clustering and comparing large sets of protein or nucleotide sequences. Bioinformatics.

[38] Hyatt D, Chen G-L, Locascio PF, Land ML, Larimer FW, Hauser LJ (2010). Prodigal: prokaryotic gene recognition and translation initiation site identification. BMC Bioinformatics.

[39] Bin Jang H, Bolduc B, Zablocki O, Kuhn JH, Roux S, Adriaenssens EM (2019). Taxonomic assignment of uncultivated prokaryotic virus genomes is enabled by gene-sharing networks. Nat Biotechnol.

[40] Cook R, Brown N, Redgwell T, Rihtman B, Barnes M, Clokie M (2021). INfrastructure for a PHAge REference Database: identification of large-scale biases in the current collection of cultured phage genomes. PHAGE.

[41] Santos-Medellin C, Zinke LA, Ter Horst AM, Gelardi DL, Parikh SJ, Emerson JB (2021). Viromes outperform total metagenomes in revealing the spatiotemporal patterns of agricultural soil viral communities. ISME J.

[42] Ettinger C, Stajich J, Coleine C (2022). Antarctic rock viral catalog.

[43] kblin/ncbi-genome-download: Scripts to download genomes from the NCBI FTP servers. https://github.com/kblin/ncbi-genome-download.

[44] Camacho C, Coulouris G, Avagyan V, Ma N, Papadopoulos J, Bealer K (2009). BLAST+: architecture and applications. BMC Bioinformatics.

[45] Edwards RA, McNair K, Faust K, Raes J, Dutilh BE (2016). Computational approaches to predict bacteriophage-host relationships. FEMS Microbiol Rev.

[46] Kothari A, Roux S, Zhang H, Prieto A, Soneja D, Chandonia J-M (2021). Ecogenomics of groundwater phages suggests niche differentiation linked to specific environmental tolerance. mSystems.

[47] Nayfach S, Roux S, Seshadri R, Udwary D, Varghese N, Schulz F (2020). A genomic catalog of Earth’s microbiomes. Nat Biotechnol.

[48] Bushnell B (2022). BBMap.

[49] Quinlan AR, Hall IM (2010). BEDTools: a flexible suite of utilities for comparing genomic features. Bioinformatics.

[50] Li H, Handsaker B, Wysoker A, Fennell T, Ruan J, Homer N (2009). The sequence alignment/map format and SAMtools. Bioinformatics.

[51] Ecogenomics/BamM: Metagenomics-focused BAM file manipulation. https://github.com/Ecogenomics/BamM.

[52] R Core Team (2021). R: A language and environment for statistical computing.

[53] Roux S, Adriaenssens EM, Dutilh BE, Koonin EV, Kropinski AM, Krupovic M (2018). Minimum information about an uncultivated virus genome (MIUViG). Nat Biotechnol.

[54] Roux S, Emerson JB, Eloe-Fadrosh EA, Sullivan MB (2017). Benchmarking viromics: an evaluation of metagenome-enabled estimates of viral community composition and diversity. PeerJ.

[55] McMurdie PJ, Holmes S (2013). phyloseq: an R package for reproducible interactive analysis and graphics of microbiome census data. PLoS ONE.

[56] Wickham H, Averick M, Bryan J, Chang W, McGowan LD, François R (2019). Welcome to the Tidyverse. J Open Source Softw.

[57] Ettinger C, Stajich J, Coleine C (2022). stajichlab/Antarctic_Virus_Discovery: v2.

[58] Turner D, Kropinski AM, Adriaenssens EM (2021). A roadmap for genome-based phage taxonomy. Viruses.

[59] Selbmann L, Onofri S, Coleine C, Buzzini P, Canini F, Zucconi L (2017). Effect of environmental parameters on biodiversity of the fungal component in lithic Antarctic communities. Extremophiles.

[60] Jarett JK, Džunková M, Schulz F, Roux S, Paez-Espino D, Eloe-Fadrosh E (2020). Insights into the dynamics between viruses and their hosts in a hot spring microbial mat. ISME J.

[61] Coleine C, Stajich JE, Pombubpa N, Zucconi L, Onofri S, Canini F (2019). Altitude and fungal diversity influence the structure of Antarctic cryptoendolithic Bacteria communities. Environ Microbiol Rep.

[62] Coleine C, Stajich JE, Zucconi L, Onofri S, Pombubpa N, Egidi E (2018). Antarctic cryptoendolithic fungal communities are highly adapted and dominated by Lecanoromycetes and Dothideomycetes. Front Microbiol.

[63] Li S, Guo R, Zhang Y, Li P, Chen F, Wang X (2022). A catalog of 48,425 nonredundant viruses from oral metagenomes expands the horizon of the human oral virome. iScience.

[64] Santos-Medellín C, Estera-Molina K, Yuan M, Pett-Ridge J, Firestone MK, Emerson JB (2022). Spatial turnover of soil viral populations and genotypes overlain by cohesive responses to moisture in grasslands. PNAS.

