# Supplementary material for: Highly diverse and unknown viruses may enhance Antarctic endoliths’ adaptability
Source: Microbiome. 2023 May 8;11:103. doi: 10.1186/s40168-023-01554-6 (PMC10165816; doi:10.1186/s40168-023-01554-6)
Supplement: Supplementary file 2 — Additional file 1: Table S1. Sample information and viral identification statistics from Antarctic metagenomes. Table S2. Final viral catalog sequence information. Table S3. DRAM-v distilled annotations for predicted phage AMGs. Figure S1. Taxonomic classification of viral clusters (VCs) that include reference genomes. Figure S2. Comparison of host predictions between MAG and RefSeq databases. [file 40168_2023_1554_MOESM1_ESM.zip › Antartic Virus - Supplemental Material - revised.pdf]

Supplementary Material for:

Highly diverse and unknown viruses may enhance Antarctic endoliths' adaptability

Cassandra L. Ettinger[a,*], Morgan Saunders[b,c], Laura Selbmann[d], Manuel Delgado-Baquerizo[e,f], Claudio Donati[g], Davide Albanese[g], Simon Roux[h], Susannah Tringe[h], Christa Pennacchio[h], Tijana G. del Rio[h], Jason E. Stajich[a,i], Claudia Coleine[a,d,e,*]

[a]*Department of Microbiology and Plant Pathology, University of California, Riverside, Riverside, California, USA.*

[b]*Department of Biological Sciences, California State Polytechnic University, Pomona, Pomona, California, USA.*

[c]*The University of Arizona, Tucson, Arizona, USA.*

[d]*Department of Ecological and Biological Sciences, University of Tuscia, Italy, Viterbo, Italy.*

[e]*Laboratorio de Biodiversidad y Funcionamiento Ecosistémico. Instituto de Recursos Naturales y Agrobiología de Sevilla (IRNAS), CSIC, Av. Reina Mercedes 10, E-41012, Sevilla, Spain*

[f]*Unidad Asociada CSIC-UPO (BioFun). Universidad Pablo de Olavide, 41013 Sevilla, Spain*

[g]*Research and Innovation Centre, Fondazione Edmund Mach, Via E. Mach 1, 38098, San Michele all'Adige, Italy*

[h]*Department of Energy Joint Genome Institute, Lawrence Berkeley National Laboratory, One Cyclotron Road, Berkeley, CA, 94720, USA*

[i]*Institute for Integrative Genome Biology, University of California, Riverside, Riverside, CA, USA.*

Correspondence to: cassande@ucr.edu, coleine@unitus.it

Supplementary Table Legends and Figures:

Table S1. Sample information and viral identification statistics from Antarctic metagenomes. Here we provide information for each Antarctic metagenome explored here including site name, geographic area, rock type, year of collection, latitude, longitude, elevation, sun exposure, distance from the sea and SRA Accession numbers. This table also reports for each metagenome the number of predicted viral sequences, the number of proviral sequences, the number of vOTUs, the number of VCs, the average viral sequence length, average number of genes per sequence and the number of viral sequences identified by CheckV as being Complete, High-quality, Medium-quality or Low-quality. Only the viral sequences in this study that met the set thresholds for inclusion based on length (i.e. ≥ 10 kbp), similarity (i.e. ≥ 95% similarity), detection (i.e. ≥ 75% of the viral genome length covered ≥ 1x by reads at ≥ 90% average nucleotide identity), and quality (i.e., exclusion of viruses with “not-determined” CheckV scores) are summarized in this table.

Table S2. Final viral catalog sequence information. Here we provide information for each viral sequence in the final catalog of 14,796 sequences including the associated sample, sequence length, proviral status, gene count, CheckV quality score, VirSorter2 predicted viral group (e.g. dsDNA phage), vOTU assignment, VC clustering status, VC assignment, taxonomy

under the old schema, taxonomy under the new proposed International Committee on Taxonomy of Viruses (ICTV) schema, and taxonomy of the predicted host. This final catalog only represents viral sequences in this study that met the set thresholds for inclusion based on length (i.e. ≥ 10 kbp), similarity (i.e. ≥ 95% similarity), detection (i.e. ≥ 75% of the viral genome length covered ≥ 1x by reads at ≥ 90% average nucleotide identity), and quality (i.e., exclusion of viruses with “not-determined” CheckV scores).

Table S3. DRAM-v distilled annotations for predicted phage AMGs. Here we provide putative AMGs, their associated viral sequence and DRAM-v distilled annotation. Only the viral sequences in this study that were predicted to belong to phage and which met the set thresholds for inclusion based on length (i.e. ≥ 10 kbp), similarity (i.e. ≥ 95% similarity), detection (i.e. ≥ 75% of the viral genome length covered ≥ 1x by reads at ≥ 90% average nucleotide identity), and quality (i.e., exclusion of viruses with “not-determined” CheckV scores) are summarized in this table.

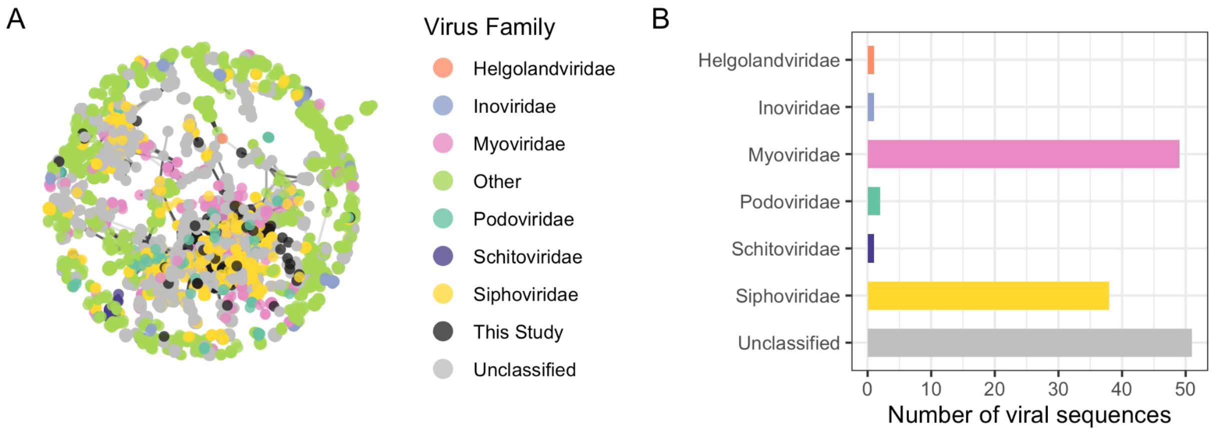

Figure S1. Taxonomic classification of viral clusters (VCs) that include reference genomes. (A) Subset of gene-sharing network from VContact2 showing only VCs that include reference genomes. Each node is a vOTU colored by predicted historical viral taxonomic family, with vOTUs identified in this study in black. Edges represent shared genes. (B) Bar chart displaying the number of viral sequences assigned to historical viral families based on VC membership with reference genomes.

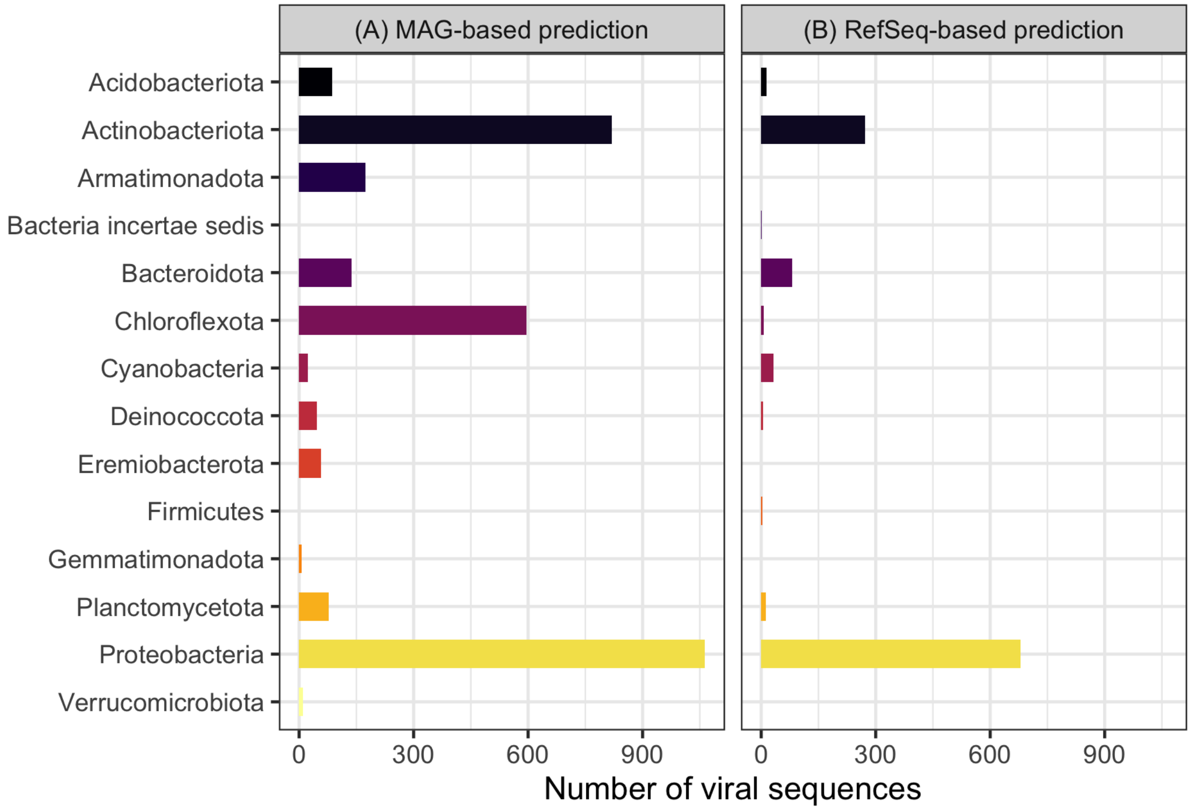

Figure S2. Comparison of host predictions between MAG and RefSeq databases. Bar charts displaying (A) MAG-based host predictions colored by predicted phylum, and (B) RefSeq-based host predictions colored by predicted phylum. Displayed are predictions for viral sequences that met the set thresholds for inclusion based on length, similarity, detection, and quality; viral sequences with no host prediction are not shown. Overall, 16.5% of viral sequences were assigned hosts based on MAG-based predictions, 3.0% were assigned hosts based on Refseq predictions, and 4.5% were assigned hosts based on both methods.
